# Identifying the Value of an eHealth Intervention Aimed at Cognitive Impairments: Observational Study in Different Contexts and Service Models

**DOI:** 10.2196/17720

**Published:** 2020-10-08

**Authors:** Monika Jurkeviciute, Lex van Velsen, Henrik Eriksson, Svante Lifvergren, Pietro Davide Trimarchi, Ulla Andin, Johan Svensson

**Affiliations:** 1 Centre for Healthcare Improvement Chalmers University of Technology Gothenburg Sweden; 2 eHealth Group Roessingh Research and Development Enschede Netherlands; 3 Skaraborg Hospital Group Lidköping Sweden; 4 IRCCS Fondazione Don Carlo Gnocchi Milan Italy; 5 Department of Internal Medicine and Clinical Nutrition, Institute of Medicine Sahlgrenska Academy University of Gothenburg Gothenburg Sweden

**Keywords:** eHealth value, evaluation of value, eHealth intervention, cognitive impairment, role of context, cost benefit

## Abstract

**Background:**

Value is one of the central concepts in health care, but it is vague within the field of summative eHealth evaluations. Moreover, the role of context in explaining the value is underexplored, and there is no explicit framework guiding the evaluation of the value of eHealth interventions. Hence, different studies conceptualize and operationalize value in different ways, ranging from measuring outcomes such as clinical efficacy or behavior change of patients or professionals to measuring the perceptions of various stakeholders or in economic terms.

**Objective:**

The objective of our study is to identify contextual factors that determine similarities and differences in the value of an eHealth intervention between two contexts. We also aim to reflect on and contribute to the discussion about the specification, assessment, and relativity of the “value” concept in the evaluation of eHealth interventions.

**Methods:**

The study concerned a 6-month eHealth intervention targeted at elderly patients (n=107) diagnosed with cognitive impairment in Italy and Sweden. The intervention introduced a case manager role and an eHealth platform to provide remote monitoring and coaching services to the patients. A model for evaluating the value of eHealth interventions was designed as monetary and nonmonetary benefits and sacrifices, based on the value conceptualizations in eHealth and marketing literature. The data was collected using the Mini–Mental State Examination (MMSE), the clock drawing test, and the 5-level EQ-5D (EQ-5D-5L). Semistructured interviews were conducted with patients and health care professionals. Monetary data was collected from the health care and technology providers.

**Results:**

The value of an eHealth intervention applied to similar types of populations but differed in different contexts. In Sweden, patients improved cognitive performance (MMSE mean 0.85, SD 1.62, *P*<.001), reduced anxiety (EQ-5D-5L mean 0.16, SD 0.54, *P*=.046), perceived their health better (EQ-5D-5L VAS scale mean 2.6, SD 9.7, *P*=.035), and both patients and health care professionals were satisfied with the care. However, the Swedish service model demonstrated an increased cost, higher workload for health care professionals, and the intervention was not cost-efficient. In Italy, the patients were satisfied with the care received, and the health care professionals felt empowered and had an acceptable workload. Moreover, the intervention was cost-effective. However, clinical efficacy and quality of life improvements have not been observed. We identified 6 factors that influence the value of eHealth intervention in a particular context: (1) service delivery design of the intervention (process of delivery), (2) organizational setup of the intervention (ie, organizational structure and professionals involved), (3) cost of different treatments, (4) hourly rates of staff for delivering the intervention, (5) lifestyle habits of the population (eg, how physically active they were in their daily life and if they were living alone or with family), and (6) local preferences on the quality of patient care.

**Conclusions:**

Value in the assessments of eHealth interventions need to be considered beyond economic terms, perceptions, or behavior changes. To obtain a holistic view of the value created, it needs to be operationalized into monetary and nonmonetary outcomes, categorizing these into benefits and sacrifices.

## Introduction

### The Concept of Value in eHealth

The concept of value has taken a central role in health care, including eHealth development and evaluation. This trend has been ongoing in health care since 2006, when Porter and Teisberg introduced the value-based health care concept to improve and innovate healthcare [1], resulting in increased interest in the *value* concept. For example, some argue that there has been a shift in the health care discourse, from improvement of quality to improvement of value [2]. Moreover, many improvements using the value concept have been reported in recent years [3]. However, the concept of value is somewhat unclear within the context of summative eHealth evaluation. Subsequently, it has been approached in different ways in various studies. Sometimes, value has been investigated as positive outcomes such as clinical efficacy or behavior change of patients or professionals [4,5]. Others have aimed to identify value through the perceptions of various stakeholders [6,7]. In order to arrange reimbursement for an eHealth service and to support decisions about investments in technology development or implementation, the value of eHealth has also been interpreted in economic terms (ie, whether health outcomes justify the costs) [8]. While different evaluation frameworks have addressed some aspects of value [9], there is no framework that explicitly guides the evaluation of value in eHealth interventions. The immature conceptual and methodological base of value in eHealth can create confusion due to the large number of studies that are hard to compare, learn from, and transfer from one context to another.

To explore the conceptualizations of value beyond the area of eHealth, inspiration could be taken from other disciplines, such as the marketing of products and services. One way to approach value can be as benefits and sacrifices, which measure service quality in relation to cost [10] and can reflect both monetary and nonmonetary outcomes [11]. In addition, value occurs from the interaction between a subject (eg, a patient) and an object (an eHealth intervention) and is relative. Relativity means that value is comparative (signifying that the value of one eHealth intervention can be compared to the other), personal (what is valuable for one end-user or stakeholder is not necessarily valuable for the other), and situational (ie, value depends on the context of use) [10].

Assuming that value is relative, one can question the usefulness of evaluating a certain eHealth intervention in a specific context. How do the results translate from one context to the next when the concept of value, costs, and benefits are potentially different? Systematic reviews that have analyzed eHealth intervention studies in dementia care [12] and eHealth evaluation frameworks [13] revealed that the role of context has been neglected. The current discourse treats the context as the circumstances under which an intervention is effective or not [14]. However, there are no studies that have explicitly investigated how the context influences the value of the eHealth intervention. Knowledge of these contextual factors can help to translate the interventions into practice or new settings [15-17].

To sum up, one way of conceptualizing the assessment of value in eHealth interventions could be to combine the benefit-and-sacrifice approach from marketing [8] with the value-based health care logic that assesses patient outcomes against cost [1]. In this view, benefits could refer to financial earnings or savings as monetary benefits, and eHealth service quality or utility as nonmonetary benefits [18]. Sacrifices relate to the financial investment and expenditure as monetary sacrifices, and social disadvantages (ie, what it takes to provide the service physically or emotionally) as nonmonetary sacrifices. In addition, it might also be fruitful to add an emphasis on the context, which has been lacking in eHealth studies [12,13,17]. The proposed structure of assessing the value of an eHealth intervention is depicted in Textbox 1.

**Structure of the value assessment of an eHealth intervention.**

**Benefits**

- Monetary
- Nonmonetary

**Sacrifices**

- Monetary
- Nonmonetary

In this study, we identified the value of a nonpharmacological eHealth intervention, which combined an integrated care model with eHealth and targeted to treat cognitive impairment in elderly populations in Italy and Sweden.

The 2 objectives that guided this study were (1) to identify the contextual factors that determined the similarities and differences in the value of an eHealth intervention between the 2 contexts, and (2) to reflect on and contribute to the discussion about the specification, assessment, and relativity of the “value” concept in the evaluation of eHealth interventions.

### Context

This study was a part of the European Union–funded project “Digital Environment for Cognitive Inclusion” (DECI). The entire DECI project was performed over 3 years and focused on the development of digital solutions to improve the care of elderly individuals with mild cognitive impairment (MCI) and mild dementia (MD).

In our study, we examined the implementation of a 6-month eHealth intervention among patients recruited in Sweden and Italy. The inclusion criteria for participants were ≥ 60 years of age, a diagnosis of MCI or a diagnosis of dementia according to DSM-5 criteria, a clinical dementia rating (CDR) of ≤ 1, living at home, and the ability to provide informed consent or the availability of a proxy for informed consent. The exclusion criteria were living in a care institution, previous or present major psychiatric illness (eg, schizophrenia, bipolar disorder, or recurrent major depression), previous or present major neurological illness other than MCI or MD (eg, stroke, multiple sclerosis, brain tumor, traumatic brain injury), the presence of other serious comorbidities (eg, severe chronic obstructive pulmonary disease, severe heart disease, or severe chronic kidney failure), a history of drug or alcohol abuse, severe sensory impairments (mainly visual and auditory), a history of intellectual disability or other developmental diseases, and a life expectancy of less than 1 year (as judged by a clinician). The specific procedures for the inclusion of patients in Italy and Sweden differed. After inclusion, the 6-month eHealth intervention was initiated.

Clinical efficacy variables and quality of life were assessed at baseline and at the end of the 6-month intervention. Further data in terms of monetary and nonmonetary outcomes were obtained by performing semistructured interviews 6 months after the eHealth intervention and by collecting information from the health care providers after the intervention had been completed.

#### The DECI Intervention

**Figure 1:**
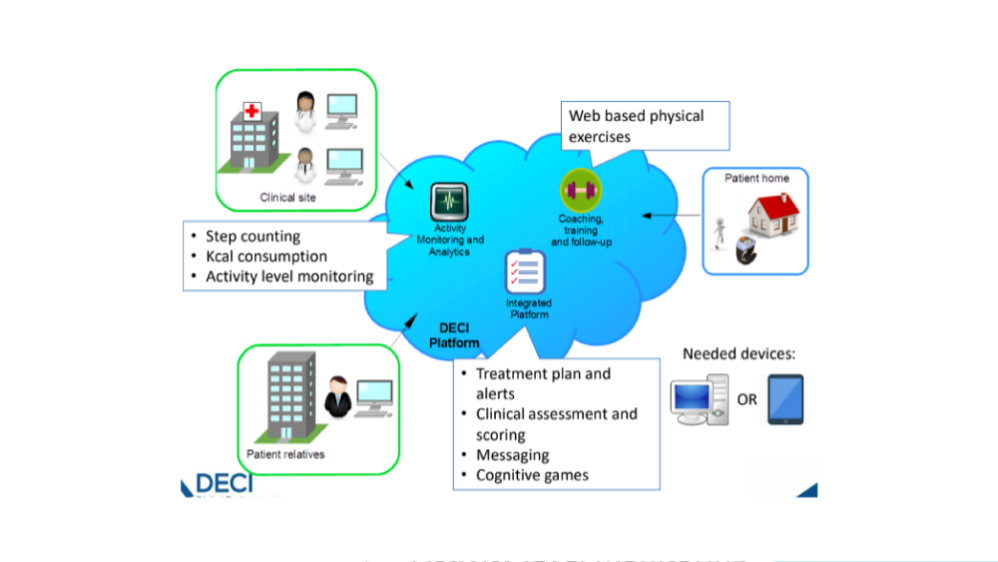
Components of the Digital Environment for Cognitive Inclusion (DECI) intervention.

The DECI intervention consisted of a set of integrated eHealth applications and a service model, as depicted in Figure 1.

**Figure 2:**
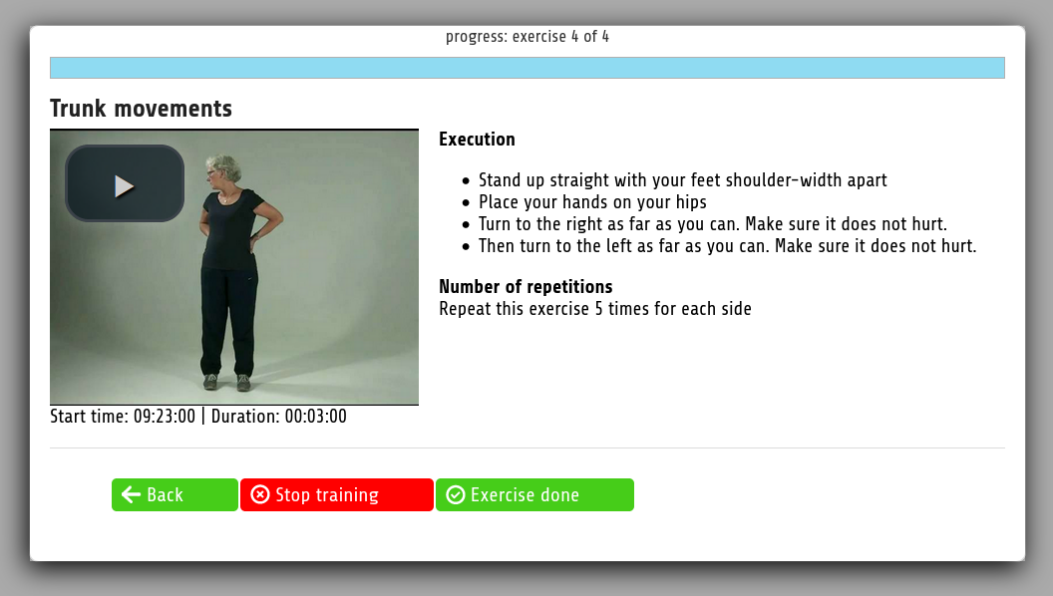
Screenshot of the Otago exercise program.

The digital component of the intervention was centered around a web-based portal that disclosed a set of several services via single sign-on. A digital platform that served as the data repository for a patient allowed for the following main functionalities: messaging among the key actors for a patient’s care (the patient, professional caregivers, family members, and informal caregivers), sending data to and retrieving data from a patient’s electronic medical record (EMR) and other digital applications, and the provision and processing of patient-centered surveys. A web-based service provided the Otago fall prevention program [19] (used for improving physical health [20]) through video instruction, accompanied by written instructions and a voice-over that pronounced these written instructions. Patients could indicate the difficulty they had with exercises; based upon this feedback, the service decided whether (1) they could continue to the next level of the Otago program after completing a week, (2) they would remain at the same level, or (3) they would go down a level. Figure 2 depicts a screenshot of an exercise in the Otago program.

Smartbrain [21], a web-based collection of exercises aimed at cognitive stimulation normally provided to patients with MCI, Alzheimer disease, Parkinson disease, and similar conditions, offered patients different difficulty levels. In addition, a physical activity dashboard supported by the ADAMO care watch (Caretek s.r.l) and that included pedometer functionality [22] allowed care professionals to inspect a patient’s physical activity (in terms of daily steps and active hours) and provided the patient with daily feedback about his or her physical activity levels.

The patients received a tablet for accessing digital services. Next, the case manager role was introduced as the organizational element of the intervention. The case manager was responsible for coordinating care and introducing the technology to the patients.

#### The Italian Context of the DECI Intervention

The Italian National Health System provides free health care for patients throughout the country. It is funded by taxes and charges for some services. The DECI study was carried out in the Milan area (Lombardy region) at the Istituto Palazzolo (IP), Fondazione Don Gnocchi (FDG). All services are integrated to offer a care program that is shared between the elderly patients and the caregivers, and to provide follow-up along all the clinical pathways. The DECI project mainly targeted patients enrolled at the Memory Clinic of the Istituto Palazzolo.

Patients eligible for inclusion were identified using the IP-FDG Memory Clinic database and were then contacted to receive information on the study and a proposal of participation. Then, an inclusion visit was arranged for written informed consent, clinical anamnesis, and a neuropsychological and functional examination of patients that accepted participation and met the inclusion criteria. Afterward, patients received training and delivery of the study materials. If needed, a follow-up visit was arranged. Lastly, clinical and satisfaction assessments were performed at the exit visit.

The Italian DECI team consisted of 1 senior physician specialized in geriatrics and dementia, 1 social worker (case manager), 2 neuropsychologists, and 1 engineer. A physiotherapist from the rehabilitation services gave advice but did not participate actively in visits and follow-up. The patients received a 10.1-inch Samsung Android tablet with a 4G SIM card to guarantee connectivity. The case manager and one of the neuropsychologists provided instructions and training for the patient regarding the DECI platform, usually during an individualized 90-minute meeting at the memory clinic. Related information was also available through the case manager by phone or through the message system of the DECI platform. The patients used the tablet for physical and cognitive training during the 6-month intervention. An online help desk by phone was established during working hours (Monday to Friday). Inclusion visits and follow-up were carried out at the memory clinic.

#### The Swedish Context of the DECI Intervention

Health care in Sweden is tax-funded and provided by the communities (elderly care) and the counties (specialized and primary care). The DECI study was carried out in the Skaraborg region, which has a permanent interorganizational network for integrated care that delivers mobile, coordinated person-centered care for patients with various chronic diseases. DECI patients receive mobile, networked care managed by the Swedish DECI team.

The study purpose was described in advertisements in local media and leaflets that were distributed in local care units. Then, patients who contacted the Swedish DECI team were invited for screening. Patients who met the inclusion criteria and agreed to participate were visited at home for written informed consent, clinical anamnesis, and neuropsychological and functional examination; inclusion and exclusion criteria were checked further. During subsequent visits, patients received training and delivery of the material. Lastly, clinical and satisfaction assessments were performed at the exit visit at the patient’s home.

The DECI team consisted of 1 senior physician specialized in geriatrics from Skaraborg Hospital Group and 1 experienced nurse from one of the communities (case manager). An occupational therapist and a physiotherapist from 2 other communities in Skaraborg gave advice but did not participate in the home visits. All patients received a 10.1-inch Samsung Android tablet with a 4G SIM card to guarantee connectivity. The patients were assessed using memory tests. The case manager and the geriatrician provided instructions and training for the patient regarding the DECI platform, usually during a 1-hour long meeting at the patient’s home. Information was also given during subsequent visits at the patients’ home by the case manager, in some cases with the geriatrician also present. The patient used the tablet for physical and cognitive training during the 6-month intervention. An online help desk by phone was established during working hours (Monday to Friday). Follow-up was carried out at the patients’ homes.

## Methods

### Value Specification

The evaluation conducted in this study was based on Textbox 1. In order to meet the requirements and aims of the DECI project, the model presented in Textbox 1 was populated with the variables collected during the project. The specification of value for the DECI intervention is depicted in Textbox 2.

**Value specification in the Digital Environment for Cognitive Inclusion (DECI) intervention.**

**Benefits**

- Monetary
  - Income
  - Prevented cost of treatment
- Nonmonetary
  - Clinical efficacy
  - Quality of life
  - Patient satisfaction
  - Job satisfaction

**Sacrifices**

- Monetary
  - Investment
  - Operating expenses
  - Cost of spent time
- Nonmonetary
  - Patient safety
  - Workload

The monetary benefits were operationalized as income and the prevented cost of treatment. The nonmonetary benefits were expressed as clinical efficacy, quality of life, patient satisfaction, and job satisfaction. The monetary sacrifices were operationalized as investment, operating expenses, and the cost of spent time. The nonmonetary sacrifices were expressed as patient safety and workload.

### Data Collection and Analysis

Data to assess the monetary and nonmonetary outcomes of DECI (Textbox 2) were collected from the Italian and Swedish health care providers involved in the project. Details of the collection and analysis of data are described below.

#### Monetary Benefits

##### Income

Income data was related to the annual state reimbursement for the treatment of MCI for a single patient. The total yearly income was calculated by multiplying the projected yearly population in the treatment by the yearly state reimbursement per patient.

##### Prevented Cost of Treatment

Prevented cost included the postponement of care and the prevented cost of treatment of falls. Postponement of care was based on the delayed conversion from MCI to MD. Data was collected using the Clinical Dementia Rating Scale (CDR) [23] at baseline and at follow-up after 6 months. The annual cost of treatment of MD was collected from the Italian and Swedish health care providers involved in the project. In the analysis of data, the conversion rate (changes in CDR after 6 months) was multiplied by the annual targeted population and the cost of MD treatment.

Data regarding falls were theorized based on the assumptions extracted from the relevant literature, since such data was not collected in the DECI study. Elderly people (from 65 years of age and older) fall 0.33 times a year [24], which can create care needs such as visits to a general practitioner (GP; 9%), emergency visits (5%) [25], or treatments for fracture (3%, based on the professional judgment of health care professionals in the DECI study). Previous studies involving the Otago physical activity coaching program have demonstrated that it helps to prevent falls by 68% [26]. The cost information for GP visits, emergency visits, and treatments for fracture was collected from the Italian and Swedish health care providers involved in the project. During the data analysis, a preventable number of falls was calculated by multiplying 0.33 by 68% and by a targeted population size. Then, a number of prevented GP visits, emergency visits, and fractures were calculated by multiplying the number of falls by 9%, 5%, and 3%, respectively. To calculate the cost, the results were multiplied by the costs for a single GP visit, emergency visit, and fracture treatment. The results were summed up to calculate the total preventable costs due to falls.

#### Nonmonetary Benefits

##### Clinical Efficacy

The Mini–Mental State Examination (MMSE) [27] and the clock drawing test (CDT) [28] were used to assess the cognitive performance of the patients. The instruments were administered at baseline (T0) and at follow-up after 6 months of intervention (T1). Within-group differences were calculated by comparing values at T1 with those at T0 using the Wilcoxon test. Between-group differences were analyzed by comparing the changes from baseline (T1–T0) using the Mann-Whitney U test. The statistical analysis was performed using SPSS for Windows (version 24; IBM Corp). A *P* value of <.05 was considered statistically significant.

##### Quality of Life

The EQ-5D-5L [29] questionnaire was used to estimate quality of life at baseline and at follow-up after 6 months. Within-group differences were calculated using the Wilcoxon test, and between-group differences were analyzed by comparing the changes from baseline using the Mann-Whitney U test.

##### Patient Satisfaction

Data were collected using semistructured interviews with patients in Sweden and Italy after 6 months of having used the DECI services services (the interview protocol can be found in Multimedia Appendix 1). The sampling was purposeful [30] in order to be careful not to create too big of a cognitive burden during the assessment activities. Thematic analysis [31] of the data helped to identify the perceived benefits by the patients and the necessary sacrifices in order to deliver it.

##### Job Satisfaction

Data were collected using in-depth semistructured interviews with the health care professionals providing the DECI services in Italy and Sweden services (the interview protocol can be found in Multimedia Appendix 2). Thematic analysis [31] of the data identified the perceived benefits by the health care professionals and the necessary sacrifices in order to deliver it.

#### Monetary Sacrifices

##### Cost of Spent Time

The spent time included the direct provision of the DECI services and participation in the multi-disciplinary meetings (those hours did not overlap). The hours spent by healthcare professionals were collected from the Swedish and Italian healthcare providers. The cost was then calculated by multiplying the number of hours by the hourly tariff. The total cost of spent time was a sum of costs of the different professional categories.

##### Investment

Investment related to the one-time cost of starting to use the DECI technologies. Investment data were collected via email from the healthcare providers.

##### Operating Expenses

Operating expenses included the annual cost of hardware (tablets), servers, the usage fees of DECI technologies, maintenance, and a help-desk function. Data were collected from the healthcare and technology providers using an Excel file containing various categories of the operating expenses. The usage fees of the DECI technology were stable, except for the fee for the ADAMO activity monitoring device (a wristwatch) that depended on the number of users.

#### Nonmonetary Sacrifices

##### Patient Safety

Data were collected using semistructured interviews with patients and health care professionals. The data were thematically analyzed in order to identify safety-related issues.

##### Workload

Data were collected through semistructured interviews with health care professionals. The data were thematically analyzed in order to identify the workload-related issues.

##### Monetary Benefit/Sacrifice Ratio

The ratio was calculated by dividing the sum of monetary benefits by the sum of monetary sacrifices (Ratio=Benefits/Sacrifices). The ratio was calculated for the scenarios at year 1, year 2, and year 3 of using the DECI services.

### Ethics

The DECI study was approved by the Ethical Committee of the Fondazione Don Carlo Gnocchi and the Regional Ethical Committee of Gothenburg.

## Results

### Demographic Characteristics

Table 1 depicts the demographic characteristics of the patients involved in the study.

**Table 1:** Demographic characteristics of patients in Italy (n=53) and Sweden (n=54) at baseline.

Characteristics		Patients in Italy (n=53)	Patients in Sweden (n=54)	*P* value^a^
Age in years, mean (SD)		77.6 (5.3)	74.8 (5.9)	<.001
**Gender, n (%)**				.66
	Female	27 (51)	30 (56)	
	Male	26 (49)	24 (44)	
**Diagnosis, n (%)**				.007
	MCI^b^	39 (74)	49 (91)	
	MD^c^	14 (26)	5 (9)	
Education years, mean (SD)		9.2 (4.3)	11.6 (2.9)	<.001
MMSE^d^ (range 0-30), mean (SD)		26.6 (2.9)	28.2 (1.4)	<.001
CDT^e^ (range 0-5), mean (SD)		3.30 (1.38)	4.81 (0.52)	<.001

^a^Differences between groups were examined using the Mann-Whitney U test for continuous variables and using chi-square tests for categorical variables.

^b^MCI: mild cognitive impairment.

^c^MD: mild dementia.

^d^MMSE: Mini–Mental State Examination.

^e^CDT: clock drawing test.

### Monetary Benefits

#### Prevented Cost of Treatment

The results in Italy showed a 10% lower conversion rate from MCI to MD compared to the conversion rates of patients in regular care. In the Swedish patients, who were younger than the Italian patients and had a less marked decrease in cognitive function at baseline (higher MMSE and CDT scores; Table 1), the results showed a 2% higher conversion rate from MCI to MD compared to the patients in regular care, thus bringing no preventable costs from the postponed care. Since the postponement of care (which is the element of the preventable costs) was zero, the Swedish preventable costs consisted of the prevention of falls only.

#### Income

The Italian and Swedish health care systems reimburse the cost of time when providing the eHealth-supported care. Therefore, the cost of time spent on providing the DECI treatment (a category in monetary sacrifices) was considered as income for year 1, year 2, and year 3.

#### Summary of Monetary Benefits

Table 2 shows a summary of the monetary benefits per patient in Italy and Sweden for 3 years.

**Table 2:** Monetary benefits, in euros (a currency exchange rate of EUR €1=US $1.18 is applicable).

Monetary benefits in Italy and Sweden		Year 1 (€)	Year 2 (€)	Year 3 (€)
**Preventable costs - Italy**				
	Total	191.696	287.544	335.468
	Per patient	1916	1916	1916
**Preventable costs - Sweden**				
	Total	6155	9232	10.770
	Per patient	12	12	12
**Income - Italy**				
	Total	67.938	101.906	118.891
	Per patient	679	679	679
**Income - Sweden**				
	Total	826.882	1.240.324	1.447.044
	Per patient	1653	1653	1653

The large differences in monetary values between Italy and Sweden occurred due to the different targeted population sizes. In Italy, it was 100 patients in year 1, 150 patients in year 2, and 175 patients in year 3. In Sweden, it was 500 patients in year 1, 750 patients in year 2, and 875 patients in year 3.

### Nonmonetary Benefits

#### Clinical Efficacy

The mean (SD) changes in MMSE and CDT scores in Italy were -0.14 (2.86) and 0.23 (1.52), respectively; in Sweden, these changes were 0.85 (1.62) and -0.11 (0.57), respectively. In Italy, both MMSE and CDT scores at the 6-month follow-up were similar to those at baseline (*P*=.35 and *P*=.34, respectively). In Sweden, MMSE scores were higher (better) at the 6-month follow-up compared to those at baseline (*P*<.001), whereas CDT scores were unchanged (*P*=.18). When comparing the results in Italy versus those in Sweden, the changes in MMSE scores were significantly greater in the Swedish cohort (*P*=.004), whereas there were no differences in CDT scores (*P*=.15). Thus, as determined by MMSE scores, cognitive performance was improved in the Swedish study population but not in the Italian study population.

#### Quality of Life

In Italy, there was no difference between the 6-month follow-up and baseline in any of the EQ-5D-5L subscales (mobility, *P*=.41; self-care, *P*=.41; activity, *P*=.58; pain, *P*=.16; anxiety, *P*=.59) and the EQ-5D-5L visual analog scale (VAS), *P*=.53. In Sweden, there were improvements in the mean (SD) changes in the EQ-5D-5L subscale anxiety and the EQ-5D-5L VAS scale, which were 0.16 (0.54) and 2.6 (9.7), respectively. The 6-month follow-up values in these variables were also significantly different from those at baseline (*P*=.046 and *P*=.035, respectively), confirming beneficial effects in these variables in the Swedish cohort. Other EQ-5D-5L subscales were unchanged in Sweden: mobility, *P*=.20; self-care, *P*=.16; activity, *P*=.20; and pain, *P*=.26. However, when comparing the results in Italy versus those in Sweden, there were no between-group differences. Therefore, although some improvement was observed in Sweden, there was no difference in the effect on quality of life between the countries.

#### Patient Satisfaction

In Italy, 10 patients were interviewed. The patients perceived the DECI service as simple and as containing engaging exercises, which helped the patients become more physically and cognitively active. The patients were willing to try more advanced exercises matching their physical condition, and it was deemed that a customized exercise program could increase the motivation of the patients. Some patients struggled to navigate the technologies and sought help from health care professionals or family members. Like in Sweden, the help-desk function could help to reduce the load on clinicians for solving technical questions or problems. The activity monitoring watch was appreciated by the patients, but its design could be improved in order to meet the aesthetic standards of the elderly.

In Sweden, 10 patients were interviewed. The DECI service was appreciated by the patients due to the clinician-monitored exercise possibilities at home and multiple home visits by health care professionals. The patients expressed a willingness to continue using the physical and cognitive activity programs. Previous information technology (IT) experience was determined to be helpful in navigating the tablet. Less experienced patients relied on family members for help, while others called health care professionals. Therefore, an IT help desk that helps to solve tablet-related issues is a necessary element of the service. The activity monitoring watch did not meet patients’ expectations without a pedometer and a display.

#### Job Satisfaction

In Italy, 4 health care professionals were interviewed. Their occupations were a geriatrician, a social worker, a neuropsychologist, and a clinical neuropsychologist. The health care professionals felt enabled to build relationships with patients through a dedicated case manager. The physical and cognitive activity coaching programs gave the professionals tools that could not be found in usual care practices. The exercises motivated and engaged the patients, which positively contributed to the job satisfaction of the professionals. The ability to remotely monitor patients’ performances and adherence to the tools was seen as an additional value. The professionals also utilized the messaging and data sharing functions in the DECI platform, which facilitated the interdisciplinary work.

In Sweden, 2 health care professionals were interviewed. Their occupations were a geriatrician and a nurse. The professionals were satisfied that the DECI services helped to form positive relationships with the patients while visiting them in their home environments. The case manager’s role was perceived as rewarding. Professionals could deepen their knowledge of the patients when observing physical status in their usual environment. The professionals felt empowered since they could offer digital tools to the patients with beneficial, customized exercises for different muscle groups and cognitive conditions. However, the messaging function in the DECI platform was perceived as having low value, since the mobile team was mostly on the road and used mobile phones for communication.

### Monetary Sacrifices

#### Investment

In Italy, the one-time investment concerned the cost of staff for server preparation in the hospital. In Sweden, the investment concerned the cost of staff for server preparation and installation of the DECI technologies. Additionally, investments in Sweden also entailed one-time costs for purchasing tablets, including 4G-sim cards for training for patients (n=52) and members of the DECI-team (n=2).

#### Operating Expenses

In both Italy and Sweden, the highest usage fee concerned the ADAMO activity monitoring device. The annual operating costs in Italy include purchasing the tablets (a stable number of tablets purchased for patients every year, depreciation in 12 months), server hosting, the configuration of the tablets, the personnel cost of maintenance and the help desk, licenses, and 4G connectivity. In Sweden, it consists of the server hosting, first-line help desk, and management costs. Operating expenses are depicted in Table 3.

**Table 3:** Monetary sacrifices, in euros (a currency exchange rate of EUR €1=US $1.18 is applicable).

Monetary sacrifices in Italy and Sweden		Year 1 (€)	Year 2 (€)	Year 3 (€)
**Cost of spent time - Italy**				
	Total	71.161	106.743	124.533
	Per patient	711	711	711
**Cost of spent time - Sweden**				
	Total	693.773	969.160	1.130.687
	Per patient	1387	1387	1387
**Investment - Italy**				
	Total	140	0	0
	Per patient	1.4	0	0
**Investment - Sweden**				
	Total	18.636	18.636	18.636
	Per patient	37	24	21
**Operating expenses - Italy**				
	Total	126.746	171.896	194.471
	Per patient	1267	1145	1111
**Operating expenses - Sweden**				
	Total	348.015	499.965	575.940
	Per patient	696	666	658

#### Cost of Spent Time

The cost was calculated based on the hours spent on the DECI service provision and the multi-disciplinary meetings (Table 3). The following professional categories were involved in Italy: physician, nurse practitioner, physiotherapist, technician, case manager (social worker), and psychologist. In Sweden, the cost was calculated for a geriatrician, an occupational therapist, a physiotherapist, and a nurse practitioner.

Table 3 shows a summary of the monetary sacrifices per patient in Italy and Sweden for 3 years. The large differences in monetary values between Italy and Sweden occurred due to the different targeted population sizes and care models.

### Nonmonetary Sacrifices

#### Patient Safety

In Italy, the ADAMO activity monitoring device caused an allergic reaction in 1 patient, due to sensitivity to nickel and plastic. The health care professionals in Italy (n=4, 1 geriatrician, 1 social worker, 1 neuropsychologist, and 1 clinical neuropsychologist) and Sweden (n=2, 1 geriatrician and 1 nurse) noted that the physical activity training program Otago could be less safe for older people if used without supervision. Hence, the level of exercise difficulty is of high importance.

#### Workload

In Italy, the 4 health care professionals dedicated, on average, 29.3 hours a week to engage with the existing patients at the memory clinic that took part in the DECI study (the result is based on their self-reported data). A substantial amount of this time was dedicated to digital data entry for the DECI study. This time was used for patient inclusion, training, phone calls, solving technical issues, and digital data entry for the study.

In Sweden, the 2 health care professionals spent, on average, 52.5 hours per week, thus reporting overtime. This time was used for the full-scale dementia examination, training, phone calls, home visits to the patients, solving technical issues, and digital data entry for the study. The result is based on their self-reported data.

### Monetary Benefit/Sacrifice Ratio

Table 4 depicts a summary of monetary benefits and sacrifices, and provides a calculation of the benefit/sacrifice ratio in Italy and Sweden for 3 years.

**Table 4:** Summary of the DECI scenario in monetary value, in euros (a currency exchange rate of EUR €1=US $1.18 is applicable).

Benefits and sacrifices in Italy and Sweden		Year 1	Year 2	Year 3
**Total monetary benefits – Italy** (€)				
	Total	259.633	389.450	454.359
	Per patient	2596	2596	2596
**Total monetary benefits – Sweden** (€)				
	Total	833.037	1.249.555	1.457.815
	Per patient	1666	1666	1666
**Total monetary sacrifices – Italy** (€)				
	Total	198.047	278.639	319.004
	Per patient	1980	1857	1822
**Total monetary sacrifices – Sweden** (€)				
	Total	1.060.424	1.487.761	1.725.263
	Per patient	2120	1983	1971
Benefit/sacrifice ratio - Italy		1.31	1.39	1.42
Benefit/ sacrifice ratio - Sweden		0.78	0.84	0.84

The benefit/sacrifice ratio showed that the Italian intervention could bring positive monetary value from the first year onward. In Sweden, the intervention did not bring monetary value during the first 3 years. However, the gap between monetary benefits and sacrifices reduces with the growing number of patients.

## Discussion

### Principal Findings

This study was guided by 2 objectives: (1) to identify the contextual factors that determine the similarities and differences in the value of an eHealth intervention between 2 contexts, and (2) to reflect on and contribute to the discussion about the specification, assessment, and the relativity of the “value” concept in evaluating eHealth interventions. This study was based on the implementation of an eHealth platform for remote home monitoring of physical and cognitive activity for people suffering from cognitive impairment in Italy and Sweden.

The findings of this study show that there is a differing value derived from the implementation of the same eHealth technology to similar types of populations in different contexts.

We translated value into benefits and sacrifices and assessed these for the intervention in 2 countries. In Sweden, the identified benefits of the eHealth intervention included improved cognitive performance assessed by the MMSE, reduced patient anxiety assessed by the EQ-5D-5L, better perceived health estimated using the EQ-5D-5L VAS scale, and satisfaction with the care received. However, these benefits can require sacrifices, such as an increased cost and higher workload for health care professionals. With the service model of Sweden (home visits), a lower number of patients could be visited per day. Additionally, the relatively high hourly rates of the staff increased the cost of the intervention. In Italy, the identified benefits included patient satisfaction with the care received, empowered health care professionals, and acceptable workloads. Moreover, for the Italian patients, who were older and had a more marked decrease in cognitive function (lower MMSE and CDT scores) at baseline than the Swedish patients, the intervention could bring positive monetary value from the first year onward. This was the result of a higher preventable cost of treatment and state reimbursement, in comparison to the cost that was based on relatively lower hourly staff rates and the service model that reduced the time spent per patient (the intervention was implemented on a sample of existing patients of the clinic and the visits were performed at the clinic). However, the clinical efficacy and quality-of-life improvements have not been observed over the course of the 6-month intervention in Italy.

In this study, the monetary side of value was influenced by factors such as (1) the service delivery design of the intervention (process of delivery), (2) the organizational setup of the intervention (ie, organizational structure and professionals involved), (3) the cost of different treatments, and (4) the hourly rates of staff for delivering the intervention. These factors affected the cost-effectiveness through the expenses incurred (including potentially preventable costs due to the intervention) and necessary investments. The nonmonetary side of the value of the intervention was also influenced by the service delivery design and organizational setup of the intervention, as well as by a fifth factor: (5) the lifestyle habits of the population (eg, how physically active they were in their daily lives and if they were living alone or with family). Finally, the value of eHealth should be seen against the sixth factor: (6) local preferences on the quality of patient care. This study showed that even the non–cost-efficient intervention can be viewed as valuable locally and deemed worthy of implementation. In such a case, local preferences on the quality of patient care can be a decisive factor. Particular positive nonmonetary outcomes might be valued highly enough to proceed with adopting the eHealth-supported service model. Moreover, it should not be neglected that the service delivery design and organizational set-up of the intervention can be adjusted to make it more cost-efficient.

A summary of the contextual factors affecting the value of eHealth intervention is provided in Textbox 3.

**Conceptual model for eHealth value specification and the influencing contextual factors.**

**Contextual factors (benefits)**

- Monetary
  - Service delivery design of an intervention (process)
  - Organizational setup of an intervention (structure)
  - Cost of different treatments
  - Hourly rates of staff for delivering an intervention
- Nonmonetary
  - Service delivery design of an intervention (process)
  - Organizational setup of an intervention (structure)
  - Lifestyle habits of the population
  - Local preferences on the quality of patient care

**Contextual factors (sacrifices)**

- Monetary
  - Service delivery design of an intervention (process)
  - Organizational setup of an intervention (structure)
  - Cost of different treatments
  - Hourly rates of staff for delivering an intervention
- Nonmonetary
  - Service delivery design of an intervention (process)
  - Organizational setup of an intervention (structure)

### Limitations

The identified contextual factors affecting the value of eHealth interventions could be limited because the study was based on a summative eHealth evaluation conducted in 2 countries. To study 2 countries in-depth demanded time and effort, and including more countries was not feasible. Other contextual setups could enrich the list of the factors and need to be investigated further. Also, the study was constrained by a 6-month follow-up time for the patients. A longer follow-up time could enrich the contextual factors and the proposed eHealth value conceptualization by revealing long-term effects.

### Comparison with Prior Work

The conceptual model proposed for assessing the value of eHealth interventions (Textbox 3) was built on prominent eHealth evaluation frameworks [32,33], in addition to previous conceptualizations of value in the eHealth [4-8] and marketing literature [10]. We argue that value needs to be operationalized in both monetary and nonmonetary outcomes, and our model suggests categorizing them into benefits and sacrifices. In practice, an evaluation study on value needs to adapt the model to its needs by populating the model with the themes of evaluation. It is important not to overlook the nonmonetary aspects that can reveal a broader and more accurate view of the value created (in contrast to the cost-versus-outcomes view [1,8,34,35]). We propose that all the parts of the model (monetary and nonmonetary benefits/sacrifices) need to be assessed in order to obtain a holistic view of the value created.

Regarding the conceptualization of value for eHealth interventions, our study showed that an overly limited view on value is obtained if assessing it as only positive outcomes, such as behavior change or clinical efficacy [4,5], perceptions regarding the added value by various stakeholders [6,7], or economic outcomes [8]. The view of value as clinical efficacy [4] is sometimes not possible when studies have a shorter follow-up time. Furthermore, this study showed that economic outcomes might be an overly limited measure of value, when the non–cost-efficient intervention can be viewed as valuable locally for other positive outcomes and deemed worthy for implementation in practice despite the increased cost. We suggest the following conceptualization of value for eHealth interventions:

Value is a holistic view of the created monetary and nonmonetary benefits of eHealth that require monetary and nonmonetary sacrifices in a particular context.

Finally, we argue that more studies need to apply a holistic view to the summative assessments of value, rather than focusing on defining value for the stakeholders during the design process [36]. This holistic value assessment could help to clarify which adjustments could be made to reduce sacrifices and maximize benefits. Moreover, such assessments (especially across multiple contexts) could improve the transferability of eHealth interventions.

### Conclusions

This study offers a step toward better conceptualization of the value concept within the eHealth context. We argue that it should be interpreted as both monetary and nonmonetary benefits and sacrifices achieved in a context. Next, we argue that without considering value beyond economic terms or assessing it as merely perceptions, a full picture of the value created might be missed. We offer a model that provides some conceptual considerations for the assessment of value of eHealth. Applied within a summative evaluation in this cross-context study, it can be a useful starting point for future research.

## Appendix

Multimedia Appendix 1 Patient interview protocol.

Multimedia Appendix 2 Healthcare professional interview protocol.

## Abbreviations

CDR: clinical dementia rating
CDT: clock drawing test
DECI: Digital Environment for Cognitive Inclusion
FDG: Fondazione Don Gnocchi
GP: general practitioner
IP: Istituto Palazzolo
IT: information technology
MCI: mild cognitive impairment
MD: mild dementia
MMSE: Mini–Mental State Examination
