# Supplementary material for: Identifying the Value of an eHealth Intervention Aimed at Cognitive Impairments: Observational Study in Different Contexts and Service Models
Source: J Med Internet Res. 2020 Oct 8;22(10):e17720. doi: 10.2196/17720 (PMC7600009; doi:10.2196/17720)
Supplement: Multimedia Appendix 1 [file jmir_v22i10e17720_app1.docx]

Appendix 1

Interview protocol with patients

| Theme | Interview questions |
| --- | --- |
| Care model | - - - 1. What did you like best in the care you received?       2. What did you like less in the care you received? |
| Overall DECI solution | - - - 1. Did you need to learn new things before you could start using the system?       2. Would you like to use DECI system in the future? (Why?/Why not?).       3. Do you think the instructions and training to use the system were sufficient? |
| Physical exercise system | - - - 1. What do you think about physical training with the help of a system? |
| Cognitive exercise system | - - - 1. What do you think about memory training with the help of a system? |
| Activity monitoring wristwatch | - - - 1. Have you tried the watch? What do you think about it?       2. How did you feel about the fact that your activity was monitored by professionals and family members?       3. Was there anything within the watch that you prefer to change (design/size/weight etc.)? |
