# Supplementary material for: Identifying the Value of an eHealth Intervention Aimed at Cognitive Impairments: Observational Study in Different Contexts and Service Models
Source: J Med Internet Res. 2020 Oct 8;22(10):e17720. doi: 10.2196/17720 (PMC7600009; doi:10.2196/17720)
Supplement: Multimedia Appendix 2 [file jmir_v22i10e17720_app2.docx]

Appendix 2

Interview protocol with healthcare professionals

| Theme | Interview questions |
| --- | --- |
| Healthcare provider characteristics | - - - 1. Your job title       2. Your occupational group: nurse, physiotherapist, geriatrician, psychiatric, occupational therapist, administrator/manager       3. How many years have you worked in your current job?       4. Your age group: 16-30; 31-50; 51+65; 66+       5. Gender: male/female       6. What is your experience with information technologies: Experienced user; Intermediate user; Basic user; No experience |
| Performance expectancy | Perceived usefulness and needs:   - - - 1. What is your overall impression of care that you provided these past months?       2. What did you like best in the care you provided?       3. What could be improved in the care process?       4. Do you think the intervention sufficiently addressed the needs of patients?       5. Did you have a responsibility of the Case Manager during intervention?       6. What do you think about having a case manager as a role in the care process?       7. What do you think about patient physical and cognitive training with the help of the system?       8. What do you think about the watch and its usefulness to patients, caregivers and healthcare professionals?   Relative advantage:   - - - 1. In your opinion, is the care process using DECI in any way better or worse compared to the usual care process for MCI and mild dementia patients?       2. How has cooperation and communication between different care units been affected during intervention? (better than before; no change; worse than before; not sure)   Job-Fit:   - - - 1. Do you think that throughout intervention you were able to do your job to a standard you are personally pleased with?       2. On average, how many hours a week have you normally worked specifically on the DECI intervention?       3. Throughout the DECI intervention, could you manage all time demands at work?       4. In your opinion, do you believe that using DECI helped you enhance your job performance? |
| Effort expectancy | Perceived ease of use:   - - - 1. Was the technology easy to understand?       2. Was the technology easy to use?       3. Did you need any technical support?       4. Do you think the instructions and training that you received in order to use the system, was enough to start using the system? |
| Perspectives | - - - 1. Would you like to use the DECI system in the future? (Discuss the reason of the answer).       2. Do you think there is a need for DECI system to be implemented in your hospital? |
| Other comments | - - - 1. Do you have any other insights or concerns regarding DECI system or intervention? (Privacy, security, suppliers, cost, etc.) |
